# Contrasting disease patterns in seropositive and seronegative neuromyelitis optica: A multicentre study of 175 patients

**DOI:** 10.1186/1742-2094-9-14

**Published:** 2012-01-19

**Authors:** Sven Jarius, Klemens Ruprecht, Brigitte Wildemann, Tania Kuempfel, Marius Ringelstein, Christian Geis, Ingo Kleiter, Christoph Kleinschnitz, Achim Berthele, Johannes Brettschneider, Kerstin Hellwig, Bernhard Hemmer, Ralf A Linker, Florian Lauda, Christoph A Mayer, Hayrettin Tumani, Arthur Melms, Corinna Trebst, Martin Stangel, Martin Marziniak, Frank Hoffmann, Sven Schippling, Jürgen H Faiss, Oliver Neuhaus, Barbara Ettrich, Christian Zentner, Kersten Guthke, Ulrich Hofstadt-van Oy, Reinhard Reuss, Hannah Pellkofer, Ulf Ziemann, Peter Kern, Klaus P Wandinger, Florian Then Bergh, Tobias Boettcher, Stefan Langel, Martin Liebetrau, Paulus S Rommer, Sabine Niehaus, Christoph Münch, Alexander Winkelmann, Uwe K Zettl U, Imke Metz, Christian Veauthier, Jörn P Sieb, Christian Wilke, Hans P Hartung, Orhan Aktas, Friedemann Paul

**Affiliations:** 1 Division of Molecular Neuroimmunology, Department of Neurology, University of Heidelberg, Heidelberg, Germany; 2 Department of Neurology, Charité - University Medicine Berlin, Berlin, Germany; 3 Institute of Clinical Neuroimmunology, Ludwig Maximilian University Munich, Munich, Germany; 4 Department of Neurology, Heinrich Heine University, Düsseldorf, Germany; 5 Department of Neurology, University of Würzburg, Würzburg, Germany; 6 Department of Neurology, University of Regensburg, Regensburg, Germany; 7 Department of Neurology, St. Josef-Hospital, Ruhr-University Bochum, Bochum, Germany; 8 Department of Neurology, Klinikum rechts der Isar, Technische Universität München, Germany; 9 Department of Neurology, University of Ulm, Ulm, Germany; 10 Department of Neurology, Friedrich Alexander University, Erlangen, Germany; 11 Department of Neurology, Goethe University Frankfurt, Frankfurt, Germany; 12 Department of Neurology, University of Tübingen, Tübingen, Germany; 13 Department of Neurology, Hannover Medical School, Hannover, Germany; 14 Department of Neurology, University of Münster, Münster, Germany; 15 Department of Neurology, Hospital Martha-Maria Halle, Halle, Germany; 16 Department of Neurology, and Institute for Neuroimmunology and Clinical Multiple Sclerosis Research, University Medical Center, Hamburg, Germany; 17 Department of Neurology, Asklepios Hospital Teupitz, Teupitz, Germany; 18 Department of Neurology, Kliniken Landkreis Sigmaringen GmbH, Sigmaringen, Germany; 19 Department of Neurology, University of Leipzig, Leipzig, Germany; 20 Department of Neurology, Klinikum Görlitz, Görlitz, Germany; 21 Department of Neurology, Klinikum Bayreuth, Bayreuth, Germany; 22 Institute of Experimental Neuroimmunology, affiliated to Euroimmun Lübeck, Lübeck, Germany; 23 Department of Neurology, Dietrich Bonhoeffer Klinikum Neubrandenburg, Neubrandenburg, Germany; 24 Department of Neurology, Rheinhessen-Fachklinik Alzey, Alzey, Germany; 25 Department of Neurology, Dr. Horst Schmidt Hospital Wiesbaden, Wiesbaden, Germany; 26 Department of Neurology, University of Rostock, Rostock, Germany; 27 Department of Neurology, Klinikum Dortmund, Dortmund, Germany; 28 Department of Neuropathology, University of Göttingen, Göttingen, Germany; 29 Department of Neurology, Hanse-Klinikum Stralsund, Stralsund, Germany; 30 Department of Neurology, Helios Vogtland-Klinikum Plauen, Plauen, Germany; 31 Neurocure, Charité - University Medicine Berlin, Berlin, Germany

**Keywords:** Neuromyelitis optica, Devic disease, Devic syndrome, longitudinally extensive transverse myelitis, recurrent optic neuritis, NMO-IgG, aquaporin-4 (AQP4) antibody, epidemiology, clinical features, magnetic resonance imaging, cerebrospinal fluid

## Abstract

**Background:**

The diagnostic and pathophysiological relevance of antibodies to aquaporin-4 (AQP4-Ab) in patients with neuromyelitis optica spectrum disorders (NMOSD) has been intensively studied. However, little is known so far about the clinical impact of AQP4-Ab seropositivity.

**Objective:**

To analyse systematically the clinical and paraclinical features associated with NMO spectrum disorders in Caucasians in a stratified fashion according to the patients' AQP4-Ab serostatus.

**Methods:**

Retrospective study of 175 Caucasian patients (AQP4-Ab positive in 78.3%).

**Results:**

Seropositive patients were found to be predominantly female (p < 0.0003), to more often have signs of co-existing autoimmunity (p < 0.00001), and to experience more severe clinical attacks. A visual acuity of ≤ 0.1 during acute optic neuritis (ON) attacks was more frequent among seropositives (p < 0.002). Similarly, motor symptoms were more common in seropositive patients, the median Medical Research Council scale (MRC) grade worse, and MRC grades ≤ 2 more frequent, in particular if patients met the 2006 revised criteria (p < 0.005, p < 0.006 and p < 0.01, respectively), the total spinal cord lesion load was higher (p < 0.006), and lesions ≥ 6 vertebral segments as well as entire spinal cord involvement more frequent (p < 0.003 and p < 0.043). By contrast, bilateral ON at onset was more common in seronegatives (p < 0.007), as was simultaneous ON and myelitis (p < 0.001); accordingly, the time to diagnosis of NMO was shorter in the seronegative group (p < 0.029). The course of disease was more often monophasic in seronegatives (p < 0.008). Seropositives and seronegatives did not differ significantly with regard to age at onset, time to relapse, annualized relapse rates, outcome from relapse (complete, partial, no recovery), annualized EDSS increase, mortality rate, supratentorial brain lesions, brainstem lesions, history of carcinoma, frequency of preceding infections, oligoclonal bands, or CSF pleocytosis. Both the time to relapse and the time to diagnosis was longer if the disease started with ON (p < 0.002 and p < 0.013). Motor symptoms or tetraparesis at first myelitis and > 1 myelitis attacks in the first year were identified as possible predictors of a worse outcome.

**Conclusion:**

This study provides an overview of the clinical and paraclinical features of NMOSD in Caucasians and demonstrates a number of distinct disease characteristics in seropositive and seronegative patients.

## Background

Neuromyelitis optica (NMO) is a severely disabling inflammatory disorder of the central nervous system (CNS) of putative autoimmune aetiology that predominantly affects the optic nerves and spinal cord [1]. NMO is associated with serum antibodies to aquaporin-4, the most abundant water channel in the CNS in up to 80% of cases [2-7]. These antibodies (termed NMO-IgG or AQP4-Ab) are thought to be directly involved in the pathogenesis of the condition [8-16]. The clinical spectrum of NMO as defined by Wingerchuk et al. (2007) [17] comprises cases of simultaneous optic neuritis (ON) and myelitis, cases of myelitis and ON, in which the two index events do not develop simultaneously but successively, and limited or inaugural forms such as single or recurrent events of longitudinally extensive myelitis (LETM) or recurrent ON [17-21]. More rarely, patients may present with brain stem encephalitis [22,23].

Similar to other autoimmune neurological diseases such as myasthenia gravis, a subset of patients exists who are seronegative. There are indications that seropositive and seronegative patients might differ with regard to clinical presentation or prognosis [24,25]. However, as AQP4-Ab were discovered only a few years ago, many previous studies investigating the clinical and paraclinical features associated with myelitis and optic neuritis in patients with NMOSD either did not determine AQP4-Ab at all [1,26], or did not stratify patients according to their AQP4-Ab serostatus [27,28], or were based on relatively small patient numbers [24,25,28-34]. Moreover, some of these previous studies did not include Caucasians [25,35] or investigated mixed non-Caucasian/Caucasian cohorts [1,36]. Finally, many previous studies were monocentre investigations and thus prone to potential selection bias.

In the present multicentre study, we aimed to analyse the clinical and paraclinical features of NMOSD in Caucasians in a stratified fashion according to the patients' AQP4-Ab serostatus.

## Patients and methods

Clinical, MRI, and CSF features from all Caucasian patients with NMOSD as defined by Wingerchuk et al. (2007) [17] and known AQP4-Ab serostatus documented in the database of the German Neuromyelitis optica Study Group (NEMOS; http://www.nemos-net.de) [37] were analysed retrospectively. All patients were seen at one of the 29 participating NEMOS centres, which included neurological departments at 17 university hospitals (Heidelberg, Berlin, Ludwig Maximilian University of Munich, Düsseldorf, Würzburg, Regensburg, Technische Universität München, Ulm, Bochum, Frankfurt, Tübingen, Hannover, Münster, Hamburg, Leipzig, Rostock, Göttingen) and at 12 academic teaching hospitals (Halle, Teupitz, Sigmaringen, Görlitz, Bayreuth, Lübeck, Neubrandenburg, Alzey, Wiesbaden, Dortmund, Stralsund, Plauen) with adjacent specialised outpatient clinics for neuroinflammatory disorders. Case ascertainment was performed between August 2009 and August 2011 by an expert panel of NEMOS members. The study was approved by the Ethics Committee of the Charité - University Medicine, Berlin, and the University of Düsseldorf, Germany, and data analysis was performed in an anonymized fashion according to the German data protection law. The Mann Whitney U test was used to test for significant differences between continuous variables and Fisher's exact test (2-tailed) to compare proportions. All tests should be understood as constituting exploratory data analysis, such that no adjustments for multiple testing have been made. Data were analysed using Microsoft Excel 2003 and GraphPad Prism 4.

At the time of analysis the database contained retrospective data from 175 Caucasian patients with NMOSD as defined by Wingerchuk et al. (2007) [17] and known AQP4-Ab serostatus. 119 patients had a history of both ON and myelitis and met Wingerchuk's 2006 revised criteria [36] (seropositive in 77.3%), 49 had a history of isolated LETM as defined by clinical myelitis and MRI lesions extending over three or more vertebral segments (seropositive in 81.6%), 7 had a history of recurrent ON (seropositive in 71.4%). AQP4-Ab was determined in a cell based assay in 55.4% [38], in a FACS based assay in 22.3% [6], in a radioimmunoprecipitation assay in 16% [7], in a fluorescence immunoprecipitation assay in 3.4% [4], and in an indirect immunofluorescence assay in 1.7% [2,3]. The median disease duration at last follow-up was 57.5 months (range, 0-390) and did not differ significantly between seropositives (60 months; range, 0-390) and seronegatives (51 months; range, 0-290). 89% had been treated with immunosuppressive or immunomodulatory agents at least once, with no significant difference between seropositive and seronegative patients (p = 0.133; Fisher exact test, 2-tailed); treatments included interferon beta (20.6%), azathioprine (45.1%), rituximab (32.6%), mitoxantrone (21.1%), oral steroids (18.9%), cyclophosphamide (12.6%), intravenous immunoglobulins (6.9%), mycophenolate mofetil (4%), methotrexate (4%), and natalizumab (2.9%). If only immunosuppressive drugs are considered, the proportion of treated patients was higher in the seropositive group (83% vs 65%; p = 0.015).

## Results

### Demographic data

The female to male ratio was 6:1 (N = 175) in the total cohort and was significantly higher among seropositive patients compared to seronegative patients (Table 1). 83.3% of the female but only 48% of the male patients were seropositive. The median age at onset was 39 years (range, 10-81) (Table 1). 24/175 patients (13.7%) were older than 60 years at disease onset, and 9 of these were older than 70 years.

**Table 1:** Comparison of demographic features according to the patients' AQP4-Ab serostatus.

	Seropositive	Seronegative	p-level
Sex ratio (male:female)			
All patients	1:10.4; N = 137	1:1.9; N = 38	p < 0.0003^‡^
Patients meeting Wingerchuk's 2006 criteria	1:9.2; N = 92	1:2; N = 27	p < 0.006^‡^
Age at onset (median, range; N)			
All patients	40 (10-81; N = 137)	38.5 (14-67; N = 38)	n.s.^§^
Patients meeting Wingerchuk's 2006 criteria	36 (10-79; N = 92)	37 (14-63; N = 27)	n.s.^§^
Cases of death, attack-related	5/137 (3.6%)°	0/38 (0%)	n.s.^‡^

°See results section for causes of death. Four additional patients died from non-attack related causes: One developed sepsis following plasma exchange; another patient with a history of autonomic dysfunction with bradycardia and recent femoral vein thrombosis died from cardiovascular failure 3 days after the second infusion with rituximab [80]; one patient died from voluntary refusal of nutrition and hydration after a severe attack of cervical myelitis that had left her tetraplegic; and one died from an accident. ^‡^Fisher's exact test (2-tailed). ^§^Mann Whitney U test. N = number of patients; n.s. = not significant.

### Disease onset

Among patients with a history of both ON and myelitis, disease started with isolated ON in 68/117 cases (58.1%), with isolated myelitis in 29 (24.8%), with simultaneous myelitis and ON in 15 (12.8%; bilateral ON in 6), and with brain stem encephalitis without concomitant myelitis or ON in 5 (4.3%). Simultaneous myelitis and ON at onset was more common among seronegative patients (Table 2). In those of them in whom the disease started with ON (with or without concomitant myelitis), ON was bilateral in 21.5% (17/79). ON was more frequently bilateral at onset in seronegative patients (Table 2).

**Table 2:** Symptoms at disease onset.

	Seropositive	Seronegative	p-level
ON at onset			
Total cohort	61/135 (45.2%)	14/38 (36.8%)	n.s.^‡^
Patients meeting Wingerchuk's 2006 criteria	56/90 (62.2%)	12/27 (44.4%)	n.s.^‡^
MY at onset			
Total cohort	64/135 (47.4%)	14/38 (36.8%)	n.s.^‡^
Patients meeting Wingerchuk's 2006 criteria	24/90 (26.7%)	5/27 (18.5%)	n.s.^‡^
BSTE at onset			
Total cohort	4/135 (3%)	1/38 (2.6%)	n.s.^‡^
Patients meeting Wingerchuk's 2006 criteria	4/90 (4.4%)	1/27 (3.7%)	n.s.^‡^
Simultaneous MY and ON at onset			
Total cohort	6/135 (4.4%)	9/38 (23.7%)	p < 0.001^‡^
Patients meeting Wingerchuk's 2006 criteria	6/90 (6.7%)	9/27 (33.3%)	p < 0.013^‡^
Bilateral ON at onset			
All patients with a history of ON	9/63 (14.3%)	9/22 (40.9%)	p < 0.015^‡^
Patients meeting Wingerchuk's 2006 criteria	9/59 (15.3%)	8/20 (40%)	p < 0.029^‡^

Data are not available for 2/175 patients. ^‡^Fisher's exact test (2-tailed). BSTE = brain stem encephalitis; MY = myelitis; n.s. = not significant; ON = optic neuritis.

### Disease course

The disease was relapsing at last follow-up in 156/175 (89.1%) patients and monophasic in 19 (ratio of monophasic to relapsing, 1:8.21). A monophasic course was more common among seronegative patients (Table 3) and, accordingly, also more frequent in patients in whom the disease started with simultaneous myelitis and ON (ratio, 1:2) compared to those in whom the disease started with either myelitis or ON (1:10.43) (p < 0.014).

**Table 3:** Comparison of clinical features according the patients' AQP4-Ab serostatus.

	Seropositive	Seronegative	p-level
Relapsing course			
Total cohort	127/137 (92.7%)	29/38 (76.3%)	p < 0.008^‡^
Patients meeting Wingerchuk's 2006 criteria	92/92 (100%)	22/27 (81.5%)	p < 0.0005^‡^
Relapse ratio, ON/MY*	0.85; N = 86	1; N = 25	n.s.^§^
Signs of-existing autoimmunity			
Total cohort	76/130 (58.5%)	3/35 (8.6%)	p < 0.00001^‡^
Patients meeting Wingerchuk's 2006 Criteria	48/86 (55.8%)	3/25 (12%)	P < 0.00009^‡^
Co-existing autoimmune disorders^†^	31/130 (23.8%)	2/35 (5.7%)	p < 0.017^‡^
Co-existing auto-antibodies only^††^	45/97(46.4%)	1/30 (3.3%)	p < 0.00001^‡^
CSF-restricted OCB at first LP			
All patients	30/110 (27.9%)	12/34 (35.3%)	n.s.^‡^
Patients meeting Wingerchuk's 2006 criteria	19/74 (25.7%)	10/26 (38.5%)	n.s.^‡^
Median CSF white cell count at first LP			
All patients	7 (0-750; N = 106)	7.5 (0-220; N = 30)	n.s.^§^
Patients meeting Wingerchuk's 2006 Criteria	7.5 (0-750; N = 68)	9 (1-220; N = 23)	n.s.^§^
Preceding infections at least once^#^	27/92 (29.3%)	5/28 (17.9%)	n.s.^‡^
Time to diagnosis of NMO (months)		11 (0-255; N = 29)	p < 0.029^§^

*Only patients with a history of both ON and MY are considered (all meeting Wingerchuk's 2006 criteria). ^† ^Diagnoses included autoimmune thyroiditis (N = 10), systemic lupus erythematosus (8), acetylcholine receptor-antibody positive myasthenia gravis (4) [74], cutaneous lupus erythematosus, Sjögren's syndrome, mixed connective tissue disorder, anti-phospholipid antibody syndrome, celiac disease, psoriasis, bullous pemphigoid, pyoderma gangrenosum, vitiligo, atopic dermatitis, sarcoidosis, HLAB27 positive ankylosing spondylitis with sacroileitis, heparin-induced thrombocytopenia, and rheumatoid arthritis. ^††^In the absence of an established autoimmune disorder; including anti-nuclear antibodies (counted only if ≥ 1:240), antibodies to double stranded DNA, antibodies to single stranded DNA, anti-histone antibodies, anti-centromere antibodies, anti-Scl70 antibodies, anti-SS/A antibodies, anti-SS/B antibodies, anti-phospholipid antibodies, MPO-specific perinuclear antineutrophil cytoplasmic antibodies (pANCA), lactoferrin-specific pANCA, pANCA of unknown specificity, thyroid peroxidase antibodies, anti-mitochondrial antibodies, anti-smooth muscle antibodies, and rheumatoid factor. ^#^Preceding the first attack or preceding relapse at least once. ^‡^Fisher's exact test (2-tailed). ^§^Mann Whitney U test. CSF = cerebrospinal fluid; LP = lumbar puncture; MY = myelitis; N = number of patients (not all data were available from all patients); n.s. = not significant; OCB = oligoclonal bands; ON = optic neuritis; WCC = white cell count.

### Time to diagnosis and time to relapse

Among patients with a history of both ON and myelitis, the correct diagnosis of NMO was made by the treating physicians after a median of 37.5 months (range, 0-390; Table 4). In 31/73 cases (42.5%) with available data, patients were misdiagnosed with multiple sclerosis (MS) by the initially treating physicians, mostly prior to the availability of NMO-IgG/AQP4-Ab testing (83.9%). A wrong initial diagnosis of MS became less common once NMO-IgG/AQP4-Ab testing became commercially available in 2005 (20% vs 54.2% before 2005) (p < 0.007). Of all patients who were initially falsely diagnosed with MS, 52.6% were treated with interferon beta (IFN-beta) at least once in the course of disease compared to 20.6% in the total cohort (p < 0.001); the frequency of IFN-beta treatment was lowest in those patients with a correct initial diagnosis (12.5%). Diagnoses other than MS, LETM, or ON were initially suspected in 11 cases, including among others acute disseminated encephalomyelitis, connective tissue disorders, and syringomyelia.

**Table 4:** Time and number of attacks until the diagnosis of NMO was made by the treating physicians.

	Time (median, range, N)	p-level
All patients	37.5 months (0-390; 112)	
ON at onset MY at onset	55 months (0-390; 66) 16 months (0-255; 29)	P < 0.013^§^
Isolated ON or MY at onset Simultaneous ON + MY at onset	49 months (0-390; 94) 1 month (0-18; 13)	P < 0.000001^§^
Seropositives Seronegatives	45 months (0-390; 88) 11 months (0-255; 24)	P < 0.029^§^

	**Number of attacks (median, range, N)**	

ON attacks MY attacks	1.0 (1-8; 118) 1.0 (1-5; 109)	n.s.^§^

^§^Mann Whitney U test. MY = myelitis; N = number of patients (not all data were available from all patients); n.s. = not significant; ON = optic neuritis.

The correct diagnosis was made earlier if the disease started with myelitis than if it started with ON (Table 4); this is partly explained by a longer median interval between first ON and first myelitis than between first myelitis and first ON (Table 5). In line with the finding that NMO started more frequently with simultaneous myelitis and ON in the seronegative group, the median time to correct diagnosis was shorter among seronegative patients (Table 4). The median number of ON and myelitis attacks until the diagnosis of NMO was made was 1.0 (range, 1-5) and 1.0 (range, 1-8) attacks, respectively. Thus, the diagnosis was not always delayed due to wrong differential diagnostic considerations (e.g. in 4 cases initially diagnosed as MS, brain MRI was always normal and oligoclonal bands never positive), but in some patients the latency between the first ON attack and the first myelitis attack (up to 349 months) or vice versa (up to 217 months) was extraordinarily long. Accordingly, in some patients a multitude of ON attacks occurred before the first myelitis took place (up to 8 events) or vice versa (up to 4 myelitis events).

**Table 5:** Time to relapse if disease started either with ON or MY.

	Time (median, range, N)	p-level
1^st ^→ 2^nd ^attack^†^		
All patients	8.5 months (1-216; N = 138)	n.a.
Patients meeting Wingerchuk's 2006 criteria	9 months (1-216; N = 97)	n.a.
Seropositives	9 months (1-201; N = 113)	n.s.
Seronegatives	5 months (1-216; N = 25)	
ON at onset	17 months (1-208; N = 67)	p < 0.002^§^
MY at onset	6 months (1-216; N = 61)	
MY+ON at onset	3.5 months (1-8; N = 10)	
Brainstem encephalitis at onset	5 months (2-20; N = 4)	
1^st ^ON → 2^nd ^ON	24 months (1-381; 79)	p < 0.0001^§^
1^st ^MY → 2^nd ^MY	7 months (0-296; 121)	
1^st ^ON → 1^st ^MY	14 months (0-349; 85)	p < 0.0005^§^
1^st ^MY → 1^st ^ON	3 months (0-217; 41)	

^†^Irrespective of whether the first event was ON or MY or simultaneous ON and MY. ^§^Mann Whitney U test. N = number of patients (not all data were available from all patients); n.a. = not applicable; n.s. = not significant.

In the total cohort, the median time between first and second event (irrespective of whether it was myelitis, ON, a combination of both, or brain stem encephalitis) was 8.5 months (Table 5). This interval did not differ significantly between seropositive and seronegative patients, but was significantly longer if the first event was ON than if it was myelitis. Similarly, the median latency between the first ON and the second ON was longer than the median latency between the first and the second myelitis. See Table 5 for details.

The difference was even more significant (p < 0.0002), if patients with isolated ON at onset were compared to all patients with symptoms other than isolated ON at onset (i.e. myelitis [6 months (1-216; N = 61], or simultaneous myelitis and ON [median, 3.5 months; range, 1-9; N = 10], or isolated brain stem symptoms [median, 5 months; range, 2-20; N = 4]).

### Relapse frequency

The median number of documented relapses per patient was 5 (range, 1-29). The ON to myelitis ratio was 0.9 among patients with NMO. Among all patients with a history of myelitis and disease duration of > 12 months, the median annual rate of myelitis attacks was 0.53 (range, 0.03-3.21). Among all patients with a history of ON and disease duration of > 12 months, a median of 0.38 (range, 0.04-3) ON attacks per year had occurred until the time of last follow-up. No significant difference regarding the annual myelitis relapse, the annual ON relapse rate, or the ON to myelitis ratio was found between seropositive and seronegative patients (see Table 3, 6 and 7). In 44/104 (42.3%) patients with NMO, myelitis and ON occurred simultaneously at least once; in another 32/60 (53.3%) the latency between myelitis and ON (or vice versa) was as short as 1-3 months at least once.

**Table 6:** Optic neuritis, comparison of clinical features according to the patient's AQP4-Ab serostatus.

	Seropositive	Seronegative	p-level
Annualized ON relapse rate^†^			
All patients with a history of ON	0.38 (0.04-2.25; N = 83)	0.36 (0.08-3; N = 21)	n.s.^§^
Patients meeting Wingerchuk's 2006 criteria	0.37 (0.04-2.25; N = 80)	0.25 (0.08-3; N = 19)	n.s.^§^
Bilateral ON ever			
All patients with a history of ON	25/92 (27.2%)	15/29 (51.7%)	p < 0.023^‡^
Patients meeting Wingerchuk's 2006 criteria	23/87 (26.4%)	14/27 (51.9%)	p < 0.019^‡^
Bilateral ON at first ON attack*			
All patients with a history of ON	12/85 (14.1%)	11/28 (39.3%)	p < 0.007^‡^
Patients meeting Wingerchuk's 2006 criteria	12/81 (14.8%)	10/26 (38.5%)	p < 0.023^‡^
ON attacks with VA ≤ 0.1			
All patients with a history of ON	83/123 (67.5%)	13/35 (37.1%)	p < 0.002^‡^
Patients meeting Wingerchuk's 2006 Criteria	74/112 (66.1%)	12/33 (36.4%)	p < 0.005^‡^
Attacks with complete recovery			
All patients with a history of ON	68/205 (33.2%)	15/51 (29.4%)	n.s.^‡^
Patients meeting Wingerchuk's 2006 Criteria	64/190 (33.7%)	13/42 (31%)	n.s.^‡^

^†^Only patients with a disease duration > = 12 months were considered. *Irrespective of whether at disease onset or not. ^‡^Fisher's exact test (2-tailed). ^§^Mann Whitney U test. ON = optic neuritis; N = number of patients (not all data were available from all patients); n.s. = not significant; VA = visual acuity.

**Table 7:** Myelitis, comparison of clinical features according to the patients' AQP4-Ab serostatus.

	Seropositive	Seronegative	p-level
Annualized myelitis attack rate^†^			
All patients with a history of myelitis	0.6 (0.03-3.21; N = 108)	0.47 (0.04-2.88; N = 27)	n.s.^§^
Patients meeting Wingerchuk's 2006 Criteria	0.51 (0.03-2.73; N = 81)	0.39 (0.09-2.88; N = 20)	n.s.^§^
Motor symptoms, first myelitis^§^			
All patients with a history of myelitis	78/120 (65%)	22/35 (62.9%)	n.s.^‡^
Patients meeting Wingerchuk's 2006 Criteria	53/81 (65.4%)	17/27 (63%)	n.s.^‡^
Motor symptoms, all attacks^§^			
All patients with a history of myelitis	293/409 (71.6%)	54/91 (59.3%)	p < 0.024^‡^
Patients meeting Wingerchuk's 2006 Criteria	226/304 (74.3%)	42/74 (56.8%)	p < 0.005^‡^
MRC grade during motor attack*			
All patients with a history of myelitis	2 (0-5; N = 82)	3 (0-5; N = 24)	p < 0.013^§^
Patients meeting Wingerchuk's 2006 Criteria	2 (0-4.5; N = 55)	3.5 (0-5; N = 18)	p < 0.006^§^
Motor attacks with MRC ≤ 2			
All patients with a history of myelitis	88/159 (55.3%)	16/48 (33.3%)	p < 0.009^‡^
Patients meeting Wingerchuk's 2006 Criteria	71/119 (59.7%)	11/33 (33.3%)	p < 0.01^‡^
Attacks with complete recovery			
All patients with a history of myelitis	55/298 (18.5%)	7/61 (11.5%)	n.s.^‡^
Patients meeting Wingerchuk's 2006 Criteria	36/223 (16.1%)	6/49 (12.2%)	n.s.^‡^

^†^Only patients with a disease duration > = 12 months were considered. *Median (range), calculated based on the patients' individual median MRC grades during all documented motor attacks. ^‡ ^Fisher's exact test (2-tailed). ^§^Mann Whitney U test. ^§^Based on 500 myelitis attacks with either (sensori)motor or sensory symptoms; 3 further documented attacks were only associated with other symptoms such as bladder/bowel dysfunction or spasticity and were therefore not included in the analysis. MRC = Medical Research Council; MY = myelitis; N = number of patients (not all data were available from all patients); n.s. = not significant.

### Accrual of disability over time

In patients with a disease duration of ≥12 months, the median annualized expanded disability status scale (EDSS) increase was 0.65 (range, 0-4.29) based on the presumption that the EDSS was 0 prior to disease onset. Importantly, the annualized EDSS progression index did not differ significantly between seropositive and seronegative patients, neither in the total cohort nor among those patients who met Wingerchuk's 2006 criteria. Also, no difference was found if the analysis was restricted to patients with a disease duration of ≥ 24, 36, 48, 60, or 100 months. Among all patients with a history of myelitis and long term follow-up (≥ 100 months), 16% (4/21) had a low EDSS progression index of < 0.2/year.

At last follow-up (median disease duration, 58 months; range, 0-390) the median EDSS was 5 (range, 0-10; N = 165). If only patients with a disease duration of 100 months or more are considered (N = 51), the median EDSS at last follow-up was 6.5 (range, 1.5-10). Although there was a slight trend towards higher EDSS values in seropositive patients compared to seronegative patients (5 vs 4), the difference did not reach statistical significance.

**Figure 1:**
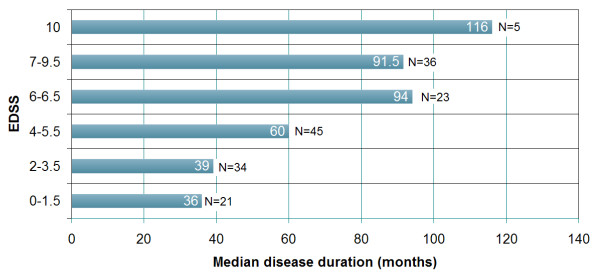
**EDSS and disease duration at last follow-up grouped according to EDSS landmarks**. EDSS < 2: No disability; EDSS 2-3.5: Disability but unrestricted walking range; EDSS 4-5.5: Restricted walking range but no walking aid required; EDSS 6-6.5: Walking aid required; 7-9.5: Essentially restricted to wheelchair or bed. In five cases the cause of death was directly related to attack-related neurological deterioration (EDSS 10). In those 4 cases in which the cause of death was not directly caused by neurological deterioration, the EDSS score obtained at the last examination prior to death was used for analysis.

**Figure 2:**
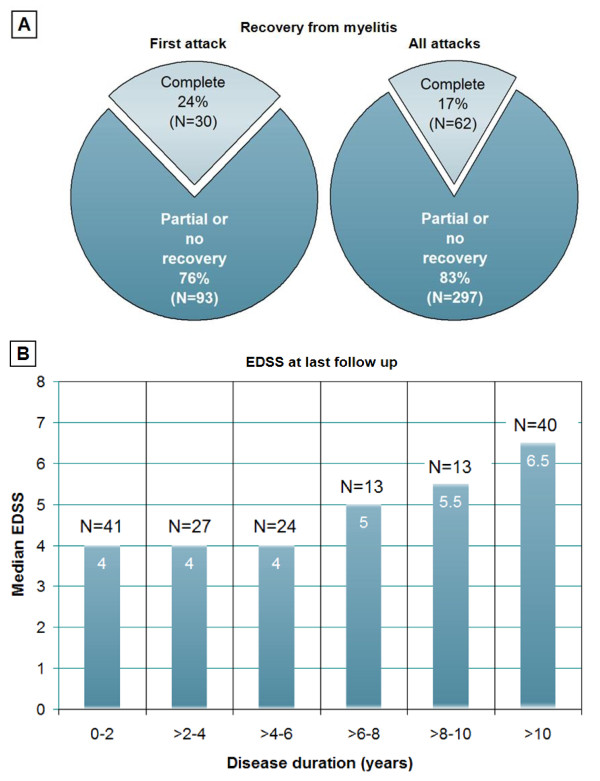
**Recovery from myelitis and median EDSS scores in patients with NMO according to Wingerchuk et al. (2006) **[36]** or LETM grouped for disease duration**. In line with the low rate of complete recovery from myelitis (A), a median EDSS of 4, indicating a restricted walking range, was notable already early in the disease course (B).

Importantly, twenty-one patients (16 seropositive, 5 seronegative; p = n.s.) had an EDSS of 1.5 or less, i.e. no disability, at the time of last follow-up; the median disease duration was 36 months (range, 5-102) in this group. See Figures 1 and 2 for additional details.

### Predictive value of early clinical events

In patients with myelitis (with or without concomitant ON) and long term follow-up data (≥ 100 months), pure sensory symptoms at onset or at first myelitis were associated with a better EDSS outcome at last follow-up compared to patients with motor symptoms at onset (Table 8). Also, tetraparesis at first myelitis and more than one myelitis attack in the first year were associated with a worse EDSS long term outcome (Table 8). By contrast, neither bilateral optic nerve involvement at first ON (irrespective if at onset or not) nor brain stem involvement (at any point in time) did predict the EDSS long term outcome.

**Table 8:** Clinical events in the early disease course and long-term outcome.

Early events in the disease course	EDSS outcome in patients with long term follow-up (≥ 100 months) (median, range; N)	p-level
Motor symptoms at first myelitis* Sensory symptoms at first myelitis*	7 (1.5-10; 29) 5.25 (2-9; 16)	p < 0.04^§^
Motor symptoms at onset Sensory symptoms at onset	8.25 (5.5-10; 8) 4 (2-6.5; 5)	p < 0.007^§^
Tetraparesis at first myelitis* Motor symptoms other than tetraparesis at first myelitis*	8.25 (7-10; 8) 6 (1.5-10; 21)	p < 0.003^§^
> 1 myelitis attacks during the first year 1 myelitis attack during the first year	8 (2.5-10; 7) 5.5 (2-8.5; 11)	p < 0.035^§^

*Irrespective of whether at disease onset or not. EDSS = expanded disability status scale; N = number of patients. ^§ ^Mann Whitney U test.

### Mortality rate and time-to-death

At the end of the observation period, 9/175 (6%) patients (all of which were seropositive) had died. In 5 of these cases the cause of death was classified as "directly related to NMO" by the last treating physicians. The exact causes of death in these patients included dyspnoea (with or without subsequent pneumonia) and cardiac arrest associated with disease relapse in three, but were not available retrospectively in two. Disease duration at the time of death was 6, 53, 116, 158, and 284 months, respectively. See Table 1 for further details.

### Myelitis - clinical findings

Motor symptoms occurred during 347/503 (69%) attacks (197 × paraparesis, 81 × tetraparesis, 31 × hemiparesis, 34 × monoparesis, 4 × Brown Sequard syndrome). The median MRC grade of all documented myelitis attacks with motor symptoms was 3 (range, 0-5). While severe paresis (MRC grade ≤2) was recorded during 49.3% of all relapses with motor symptoms (including MRC grade 0 in one or more limbs in 19.7%), paresis was mild (MRC grade 4 or 5-) in 31%. Severe paresis was more frequent in the seropositive group than in the seronegative group (Table 7); accordingly, the median of all patients' individual median MRC grades was worse in the seropositive group (Table 7). Exclusively sensory symptoms were recorded during 153 myelitis attacks (29.9%); dysaesthesia or pain were explicitly mentioned in 46 cases. Pure sensory attacks were more common in the seronegative group and, accordingly, motor symptoms more common in the seropositive group (Table 7).

13 patients developed dyspnoea at least once over the course of disease (seropositive in 11; seronegative in 2). Other symptoms included ataxia, bladder and bowel disturbances, erectile dysfunction, and Lhermitte's sign.

In those with available data in whom the disease started with myelitis (with or without concomitant ON), motor symptoms were among the presenting symptoms in 55/83 (66.3%), with tetraparesis (with dyspnoe in 1 case) being present in 15 of them. The median MRC grade in this group was 3 (range, 0-5; N = 37); 14 of these patients (37.8%) presented at onset with severe paresis as indicated by an MRC grade ≤ 2.

### Myelitis - MRI findings

**Figure 3:**
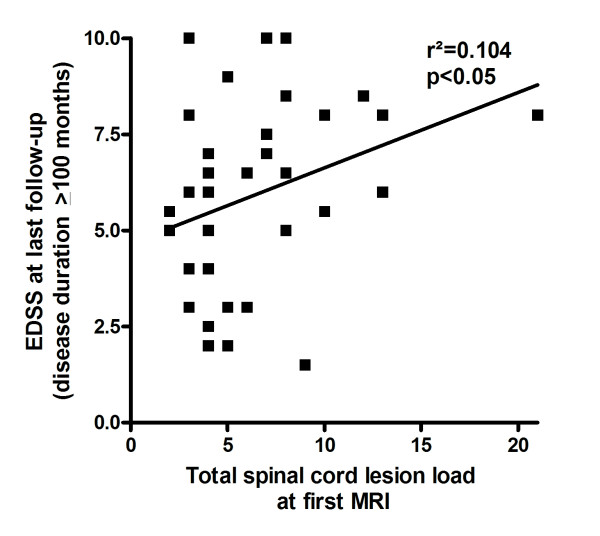
**Correlation between the total spinal cord lesion load at first myelitis as measured by MRI and the EDSS at last follow-up in patients with a disease duration of at least 100 months**.

At first myelitis, MRI showed at least one spinal cord lesion extending over 3 or more vertebral segments in 127/137 patients with available data (92.7%). The median extension was 6 segments (range, 1-21; N = 137) with a non-significant trend towards longer lesions in seropositives (Table 9). In 21 patients (18 seropositive), a second lesion was detected (median extension, 2 segments; range, 1-8), and in 8 patients (all seropositive) an additional third lesion (median, 1 segment; range, 1-3) was present. In those patients with several lesions, the median total number of segments involved was 8 (range, 2-21). The total lesion load at first MRI correlated with the EDSS at last follow up in patients with a disease duration > = 100 months (Figure 3). The spinal conus was involved in at least 4 patients. 37 patients had lesions in the cervical portion of the spinal cord, 27 in the thoracic portion, and 44 in both the cervical and the thoracic portions. Lesions involved the lumbar or sacral spinal cord in addition to the cervical or thoracic portions in only 15 patients.

**Table 9:** Myelitis, comparison of spinal cord MRI features according to the patients' AQP4-Ab serostatus.

	Seropositive	Seronegative	p-level
Median lesion length^†^, first MRI	6 (1-21; N = 104)	4.5 (2-12; N = 26)	n.s.^§^
Median lesion length^†^, all MRIs	5 (1-21; N = 264)	4 (1-16; N = 47)	p < 0.01^§^
SC lesions ≥ 3 vertebral segments, 1^st ^MRI	134/142 (94.4%)	38/40 (95%)^#^	n.s.^‡^
SC lesions ≥ 3 vertebral segments, all MRIs	240/274 (87.6%)	46/51 (90.2%)^#^	n.s.^‡^
SC lesions ≥ 6 vertebral segments, 1st MRI	63/115 (54.8%)	10/28 (35.7%)	n.s.^‡^
SC lesions ≥ 6 vertebral segments, all MRIs	131/264 (49.6%)	12/47 (25.5%)	p < 0.003^‡^
Patients with entire SC involvement at least once	15/132 (11.4%)	0/36 (0%)	p < 0.043^‡^
MRIs with more than one lesion	59/274 (21.5%)	9/51 (17.6%)	n.s.^‡^
Total lesion load*, first MRI	6.5 (1-21; 104)	5 (2-12; 26)	p < 0.022^§^
Total lesion load*, all MRIs	6 (1-21; 264)	5 (1-16; 47)	p < 0.006^§^
Total lesion load > 6 segments*, first MRI	65/104 (62.5%)	11/26 (42.3%)	n.s.^‡^
Total lesion load > 6 segments*, all MRIs	153/264 (58%)	17/47 (36.2%)	p < 0.007^‡^

Values are given as median (range, number) or as proportions. ^†^If more than one lesion was present in the same MRI, the longest lesion was counted. *Median cumulative length of all lesions present in the same spinal cord MRI. ^#^Note that all seronegative patients with NMO according to Wingerchuk 2006 or LETM had lesions extending over 3 or more vertebral segments at least once *per definitionem*. ^‡^Fisher's exact test (2-tailed). ^§ ^Mann Whitney U test. MRI = magnetic resonance imaging; N = number of patients (not all data were available from all patients); n.s. = not significant; SC = spinal cord.

If all 326 spinal MRIs are considered, the median length of the longest lesion in each MRI was 5 segments (range, 1-21) and was slightly longer in seropositive patients than in seronegative patients (Table 9). 286 MRIs (87.7%) showed lesions that ranged over 3 or more segments. In 68 MRIs, a second lesion was detectable (median, 2 segments; range, 1-8), and in 19 of them a third lesion (median, 1 segments; range, 1-3); the median total spinal cord lesion load of all MRIs was higher in the seropositive group (Table 9). Very long lesions extending over 6 or more segments were more frequent among seropositives, and entire spinal cord involvement as defined by contiguous spinal cord lesions extending ≥ 17 vertebral segments occurred only in seropositive patients (Table 9).

### Myelitis - outcome

Myelitis attacks were mainly treated with intravenous methylprednisolone (378 attacks) and/or plasma exchange (78 attacks). Other treatments included oral prednisolone, IVIG, and rituximab. Complete remission was reported in 62/359 (17.3%), partial remission in 248 (69.1%), and no remission in 49 (14%). The proportion of attacks with only partial or no recovery was lowest at the time of the first attack and increased with the number of subsequent attacks (first attack, 75.6%; second, 87%; third: 85.7%; fourth, 79.5%; fifth, 81.8%; sixth, 100%; seventh, 88.9%; eighth, 100%; ninth, 100%; tenth, 100%) (Figure 2). No significant difference regarding attack outcome (complete, partial, or no recovery) was found between seropositive and seronegative patients (Table 9). In 12/14 (85.7%) of those cases with severe paresis (MRC grade ≤ 2) at onset, no or only partial remission was achieved.

### Optic neuritis - clinical findings

During the first documented ON attack, visual acuity was ≤ 0.1 on either the left or the right eye in 30/39 (76.9%) patients with available data. In total, results from 158 eye examinations during acute ON were available for analysis. 96 examinations (60.8%) in 62 patients showed a visual acuity of ≤ 0.1 in the affected eye(s), which was more frequent among seropositive than among seronegative patients (Table 6).

At the end of the observation period, both the right and the left eye had been affected at least once (either simultaneously or successively) over the course of disease in 64% (78/121) of patients with a history ON and in 76.1% (35/46) of those with long term follow-up (≥ 100 months); simultaneous affection of both eyes occurred at least once in 33.1% and 32.6%, retrospectively. If all documented ON attacks are considered, ON was bilateral in 19.8% or 62/251 attacks.

### ON - outcome

ON attacks were mainly treated with intravenous methylprednisolone (N = 198) alone or in combination with plasma exchange or immunoadsorption (N = 30). Other treatments included oral prednisolone, IVIG, and rituximab. Complete remission was reported in 83/256 ON attacks (32.4%), partial remission in 126 (49.2%), and no remission in 47 (18.4%). No differences regarding outcome were observed between seropositive (no or only partial recovery in 66.8%) and seronegative patients (70.6%; Table 6). Complete remission was rare already at disease onset; in 22/29 patients (75.9%) no or only partial remission was achieved.

### Brain stem lesions

In 46/175 patients (26.3%), clinical and/or radiological signs of brain stem involvement were recorded at least once over the course of disease, with no significant difference between seropositive and seronegative cases (Table 10). In 15 of these patients, all of whom were positive for AQP4-Ab, brain stem lesions occurred independently of spinal cord or cerebral symptoms at least once. Attack-related hiccups and/or vomiting, indicating involvement of the medulla oblongata, were noted in 14 patients (all seropositive) during acute relapse. Other brain stem symptoms observed included dysphagia, oculomotor dysfunction (internuclear opthalmoplegia, abducens nerve palsy, and nystagmus), ptosis, laryngeal spasm, and tonic brain stem attacks. Although the medulla oblongata was the most common site of infratentorial lesions, lesions were also detected in the pons, cerebellar peduncles, mesencephalon, and diencephalon (bithalamic in one seropositive patient) in some patients. Overall, 69 attacks involving the brain stem were documented.

**Table 10:** Frequency of supratentorial and infratentorial brain involvement.

	Seropositive (No of patients, %)	Seronegative (No of patients, %)	p-level
Supratentorial brain lesions at first MRI			
Total cohort	63/133 (47.4%)^†^	16/35 (45.7%)^†^	n.s.^‡^
Patients meeting Wingerchuk's 2006 criteria	38/89 (42.7%)	13/26 (50%)	n.s.^‡^
Supratentorial brain lesions ever			
Total cohort	78/133 (58.7%)	21/36 (58.3%)	n.s.^‡^
Patients meeting Wingerchuk's 2006 criteria	51/89 (57.3%)	16/27 (59.3%)	n.s.^‡^
Brain stem involvement^#^			n.s.^‡^
Total cohort	39/137 (28.5%)	7/38 (18.4%)	n.s.^‡^
Patients meeting Wingerchuk's 2006 criteria	31/137 (22.6%)	7/38 (18.4%)	n.s.^‡^

^†^Considered "compatible with MS" by the reading radiologists in 11 cases (8.9%) in the seropositive group and in 3 cases (9.4%) in the seronegative group (p = n.s.). ^#^Clinical and/or MRI evidence. ^‡^Fisher's exact test (2-tailed).

### Supratentorial brain lesions

The first available brain MRI showed supratentorial brain lesions in 81/168 (48.2%) cases. Lesions were classified as "compatible with a diagnosis of MS" in 14/81 patients (17.5%) by the treating radiologists, and as "non-MS specific" in the remaining cases, with no difference between seropositives and seronegatives (Table 10). Considering not only the first but all MRIs available for analysis, supratentorial lesions were detectable in 99/168 patients (58.6%) at least once and in 179/351 MRIs (51%). While 29/178 (16.3%) were classified by the reading radiologists as showing lesions "compatible with MS", 150 were classified as showing "non-MS specific" brain lesions. Only 3 out of 57 patients (5.3%) who had no supratentorial lesions at first examination, and 5 out of 34 (14.7%) patients who had non-MS specific supratentorial lesions developed lesions classified as "compatible with the diagnosis of MS" later in the disease course; AQP4-Ab were positive in all of these cases. In addition, 15 out of 57 patients (26.3%) who had a normal first supratentorial brain MRI developed "non-MS-specific" supratentorial lesions later on (seropositive in 12). In the remaining cases, no follow-up brain MRI was performed.

### CSF findings

At first lumbar puncture (LP), cerebrospinal fluid (CSF) restricted oligoclonal bands (OCBs) were present in 42/144 patients (29.2%) with no significant difference between seropositives and seronegatives (Table 3). Follow-up results were available from 83 patients (65 from initially OCB-negative patients and 18 from initially OCB-positive patients). 10/65 patients that were negative for OCBs at first LP (15.4%) developed OCBs later, and 10/18 patients positive for OCBs at first LP (55.6%), turned negative at follow-up. Consequently, 52/144 (36.1%) patients were positive for OCBs at least once. The intrathecal, polyspecific humoral immune response to measles, rubella, and varicella zoster (so called 'MRZ reaction') [39], a marker of classical MS, was tested in 18 patients (seropositive in 13) but was negative in all of them (7 of whom have previously been reported in [40]).

CSF white cell numbers at first lumbar puncture were rather low (median 7/μl; range, 0-750; N = 136). CSF pleocytosis, as defined by a white cell count (WCC) > 5/μl, was present at first LP in 95/146 patients (65.1%; median, 18 cells/μl; range, 6-750). The frequency of pleocytosis as well as the median WCC did not differ between seropositive and seronegative patients (Table 3). CSF neutrophils were documented in 29 cases (median, 10% of all white cells; range, 1-93.33%; seropositive in 25); however, cytological results were not available from most patients.

### Preceding infections

Attacks were reported to have occurred following acute infection in 32 patients (Table 3). In 9 cases (one of whom was previously reported [41], acute herpes virus infection (herpes zoster in 5; herpes facialis in 1; "cutaneous herpes infection" in 1) preceded the first attack. One patient was first diagnosed with active tuberculosis of the lung at NMO onset. Other preceding events included "common cold" and "feverish infection" (N = 10); sinusitis (2); otitis media (1); bronchitis (1); pneumonia (1); urinary tract infection (7; including urosepsis in 1); and gastrointestinal infection (2).

### Co-existing autoimmune disorders

Co-existing autoimmune disorders were present in 33/165 (28.1%) patients; and in 46 additional cases, autoantibodies (but not clinical signs) usually associated with autoimmune disorders or indicating systemic autoimmunity were reported (see legend to Table 3 for details). Signs of co-existing autoimmunity were significantly more frequent in the seropositive group (Table 3).

### Other co-existing diseases

Seven patients (4%; all seropositive) had a history of cancer, including breast cancer (3 cases), cervical cancer, rectal carcinoma, anal carcinoma, vulvar carcinoma, nasopharyngeal carcinoma, and skin cancer. Two additional patients were diagnosed with a monoclonal gammopathy. Benign tumour diagnoses present in this cohort included meningeoma (2 cases), fibroadenoma of the breast, uterus myoma, and hepatic hemangiomas. In three further patients hysterectomy and/or ovarectomy for unspecified reasons had been performed. At least 21 patients had a history of severe and/or chronic infectious diseases, including hepatitis C, hepatitis B, hepatitis A, tuberculosis, borreliosis, HPV (1 × condylomata accuminata, 1 × verrucae planae), herpes zoster, herpes labialis and herpes genitalis, meningitis, osteomyelitis, pyelonephritis, salpingitis, and coxsackievirus infection.

### Family history

In two seropositive cases, the patient's father had reportedly suffered from "multiple sclerosis". A chronic inflammatory CNS disease was also suspected in the grandmother and in the brother, respectively, of two further seropositive patients. Nine AQP4-positive patients reported a family history of non-CNS autoimmunity including rheumatoid arthritis (N = 7; including the mother of the two patients with paternal MS), systemic lupus erythematosus (SLE; 2), and diabetes mellitus (2). Some of these patients were diagnosed with co-existing autoimmune disorders themselves (including SLE, myasthenia gravis, autoimmune thyroiditis, and arthritis).

## Discussion

In this study we analysed the clinical and paraclincal features associated with myelitis and optic neuritis in a large cohort of Caucasian patients with neuromyelitis optica spectrum disorders in a stratified fashion according to the patients' AQP4-Ab serostatus. Clinically, the disease course was characterized by severe attacks in many patients despite immunosuppressive and immunomodulatory treatment, often resulting in disability and impaired mobility within short time. More than two thirds of all myelitis attacks were associated with motor symptoms (with sensorimotor paraparesis as the most common single manifestation), and around half of all motor attacks resulted in an MRC grade of 2 or lower in one or more limbs. Every fourth motor attack was associated with tetraparesis, leading to respiratory failure in some cases, and in more than a quarter of patients, the disease was not restricted to the spinal cord but involved the brain stem as well. The rate of no or incomplete recovery from myelitis was high (around 75%) already from the beginning, and worsened with the number of subsequent relapses. Accordingly, around 40% of all patients with a history of myelitis had an EDSS of 6 or higher at the end of the observational period. Similarly, the visual deficiency present during acute ON attacks was often severe (visual acuity ≤ 0.1 in more than 60% of all relapses), and remission was incomplete in most cases. Motor symptoms at onset, tetraparesis early in the disease course, and more than one myelitis attack during the first disease year were identified as possible predictors of a worse clinical outcome in patients with long term follow-up. The median number of relapses per year (0.53 myelitis attacks and 0.38 ON attacks) was similar to that observed in patients with MS under standard treatment with IFN-beta (0.29-1.82) or glatiramer acetate (0.34-1.19)[42], but the time to EDSS 6-6.5 (walking aid needed) was much shorter (7.8 years) than that noted even in untreated exacerbating-remitting MS patients (23 years) [43]. The latter finding is likely to reflect both the preferential affection of the spinal cord in NMO as compared to MS and the differential modes of inflammatory tissue damage in the two conditions. While MS primarily causes demyelination, NMO attacks are often associated with severe necrosis [44]. Seropositive and seronegative patients were found to differ with regard to attack severity and clinical presentation. Visual acuity of ≤ 0.1 during acute ON attacks was more frequent among seropositives, motor symptoms were more common in seropositive patients, the median MRC grade during acute myelitis attacks worse, and MRC grades ≤ 2 more frequent, in particular if patients met Wingerchuk's criteria [17]. On the other hand, simultaneous myelitis and ON as well as bilateral ON at disease onset, and sensory symptoms were more frequent in the seronegative group. By contrast, the two groups did not differ significantly with regard to the median annualized total relapse rate, the median annualized myelitis specific relapse rate, the median annualized ON specific relapse rate, relapse outcome (no remission, partial or complete remission), and the frequency of brainstem involvement.

Given both the low remission rate (as compared to MS) found in our study already at disease onset and the fact that the first event was followed by a relapse after a median latency of just 9 months (and only 5 months in the seronegative group), early treatment and, therefore, an early diagnosis of NMO is crucial. However, this study revealed a marked delay in the diagnosis of NMO (16 months from onset if the disease started with myelitis, and even 55 months if the disease started with ON; p < 0.013). Most importantly, a substantial number of patients (42.5%) were initially wrongly diagnosed with MS, and 52.6% of these patients were treated at least once with IFN-beta, a drug which is safe and effective in MS but considered to be harmful in NMO [45-50]. In addition to incorrect differential diagnostic considerations on the part of treating physicians, other factors may have also contributed to this delay. In some patients the latency between the first ON attack and the first myelitis attack or vice versa was extraordinarily long. Also, the presence of supratentorial brain lesions, which has only recently been recognized as a common feature of NMO [36,51,52], as well as the fact that short MRI lesions not exceeding two vertebral segments and/or positive OCBs, a hallmark of MS, were present at onset in some patients, might have played a role. The median time to diagnosis was shorter among seronegative patients (11 months) than among seropositive patients (45 months), which is partly explained by the fact that NMO started more frequently with simultaneous myelitis and ON in the seronegative group.

From a diagnostic point of view, the following findings are of particular importance.

First, mild symptoms or a benign long-term course do not rule out the diagnosis of NMO, but occur in a substantial number of (both seropositive and seronegative) NMO cases. While the concept of benign MS is now well recognized [53], only a few cases of benign NMO have been reported thus far [54-56]. It is therefore of interest that around 17% of patients with long-term follow-up (≥ 100 months) in the present cohort were fully ambulatory (EDSS < 4) at last examination, including one (seropositive) case with an EDSS of 1.5 (indicating minimal neurological signs, but no disability) after 102 months. Similarly, a considerable number of myelitis attacks were either purely sensory (around 30%) or associated with only mild paresis, especially in seronegatives.

Second, a subset of patients (irrespective of the AQP4-Ab serostatus) may present with rare manifestations not typically associated with NMO. While the broad majority of patients presented with tetraparesis and paraparesis in our cohort, in a minority of cases hemiparesis, Brown Sequard syndrome, or monoparesis may occur, even at disease onset. NMO attacks may also exclusively affect the brain stem, independent of myelitis or optic neuritis, as observed in 15 cases in our series. Isolated brain stem encephalitis may even be the presenting symptom, as was the case in 5 of our patients. One of these patients, a young girl, was initially falsely diagnosed with bulimia nervosa because of repeated vomiting, others with gastro-intestinal infection. Moreover, besides intractable vomiting and hiccups, which have been repeatedly described in NMO [23,57,58], other brain stem symptoms such as dysphagia, laryngeal spasm, and oculomotor dysfunction occurred in a subset of patients and do not rule out the diagnosis of NMO as demonstrated here. Accordingly, brainstem lesions as detected by MRI were not in all cases restricted to the medulla oblongata and to the diencephalon, which are known sites of predilection in NMO [59], but may also occur in the pons, the cerebral peduncles, and the cerebellar peduncles.

Third, most patients did not present with simultaneous myelitis and bilateral ON, the classical syndrome described by Devic. Instead, NMO started in most cases with unilateral ON and, more rarely, with isolated myelitis. Importantly, the first ON attack was followed by myelitis after a median of just 14 months in the seropositive group, and the first myelitis was followed by ON after a median of only 3 months. This corroborates findings from previous, smaller studies, which found that AQP4-Ab seropositivity in patients with isolated ON or myelitis confers a high risk of conversion to NMO within one year [18-21] and strongly underlines the need for early prophylactic treatment in patients presenting with seropositive isolated ON or myelitis.

We found a strong female preponderance in our cohort. Interestingly, the female/male ratio was considerably higher than that observed in MS (1:2) [60] in the seropositive group (10.4), which is in accordance with a smaller previous study [61], but was similar to MS in the seronegative group (1.9). This finding further supports the concept that seropositive NMO is a distinct entity, different from classical MS and seronegative NMO. In line with the latter presumption, the median age of onset (40 years) in the seropositive group was higher by a decade than that in classical MS (29 years) [60].

The mortality rate was much lower in the present cohort (6% after a median observation time of 57.5 months) than that in a previously published North American cohort, which reported a five-year survival rate of only 68% [1]. This may partly reflect an increase in awareness of the condition and thus earlier treatment. While the median year of onset in our study was 2004 (range, 1977-2011), in that previous study disease had started in 1985 in the relapsing subgroup, and in 1977 in the monophasic subgroup. The difference in mortality rates might also be the result of improved treatment options. Fifty-seven patients in our cohort were treated with rituximab at least once over the course of disease, and 37 with mitoxantrone, drugs that are now considered to be effective in NMO, but which were not available in the 1980s and 1990s when most of the patients reported by Wingerchuk and colleagues were seen. Similarly, testing for NMO-IgG/AQP4-Ab, which largely contributes to the laboratory differentiation of NMO and classical MS and which can thus guide treatment decisions, only became available in the middle of the last decade. Finally, genetic factors might also play a role. While around 40% of the patients in the North American series were classified as 'non-white' by the authors, our cohort consisted of an exclusively Caucasian population. Importantly, only 5 of the 9 patients who died during the observation period in our study died from attack-related neurological complications.

Recent studies have revealed a high frequency of brain lesions in Asian and mixed Asian/Caucasian NMO cohorts. Our data corroborate this finding in an exclusively Caucasian cohort. Supratentorial brain lesions were detectable in around 50% of all patients at first examination, irrespective of the AQP4-Ab antibody status, both in the total cohort and among those patients who met Wingerchuk's criteria, and at least once over the course of disease in 60.1%. The fact that around 17.7% of these MRIs were classified as "compatible with MS" by the reading radiologists underscores the relevance of AQP4-Ab, which was positive in 78.6% of these patients, in the differential diagnosis of MS and NMO.

Besides AQP4-Ab, the length of the spinal cord lesions is thought to discriminate between NMO and MS with high specificity [36]. In fact, the first MRI showed at least one longitudinally extensive lesion in the vast majority of our patients. Importantly, however, 7.3% had a lesion shorter than three segments at first examination. Such cases can pose a serious differential diagnostic challenge (even more so as 60% had brain lesions on their first MRI). In 8 of these patients, the diagnosis of NMO could be established based on a positive AQP4-Ab test result. In the seronegative ones, the diagnosis of NMO could only be made based on the finding of lesions spanning over ≥ 3 segments on a follow-up spinal MRI later in the disease course and a negative brain MRI at onset. This underlines the importance of early AQP4-Ab testing also in patients presenting with myelitis and short spinal cord lesions as well as a need for repeated spinal cord imaging in these patients.

In the three largest previous studies [1,27,36], spinal cord lesions were reported to have spanned over 3 or more vertebral segments in most patients, but the exact length was not stated. In our cohort, spinal lesions had a median length of 6 segments, which is normally not found in MS [36]. In addition, we evaluated whether more than one spinal cord lesion were present, and if so, calculated the total lesion load. In fact, 21 patients had an additional second or even a third lesion already at first examination, some of which also extended over three or more segments, corresponding to a median total lesion load of 8 vertebral segments. Again, such high lesion load is very unusual in classical MS. In line with the finding of more severe myelitis in the seropositive group, the total spinal cord lesion load was also higher among the seropositives in our cohort, lesions > = 6 segments were more frequent, and involvement of the entire spinal cord was observed only among seropositives.

At the time of first MRI examination, most patients had lesions located both in the cervical and thoracic portions of the spine. Among those with lesions restricted to either of the two sites, cervical involvement was slightly more common. However, 15 patients had additional lesions of the lumbar portion and/or the spinal conus. Together with our finding of multiple lesions in a considerable subset of patients, these data demonstrate that MRI examinations should not be restricted to the cervical spine but should ideally include the entire spinal cord.

The diagnosis of NMO according to Wingerchuk et al. (2006) [36] could be made based on the presence of two index events and brain and spinal cord MRI findings alone in 79% of all NMO patients and in 74.4% of the seropositive ones (not shown). Conversely, either brain MRI or spinal cord MRI was not formally required in 25.6% of the AQP4-Ab positive NMO patients to establish the diagnosis. However, we do not recommend dismissing brain and spinal cord imaging in seropositive cases, all the more as fulfilment of all three supportive criteria has been demonstrated to improve the specificity of the 2006 diagnostic criteria [36].

CSF findings in NMO are known to differ significantly from those in classical MS [62-64]. In accordance with previous studies, OCBs were present in only one third of our patients (compared to > 95% in MS) [65,66]. Importantly, more than half of patients positive for OCBs at first LP became negative at follow-up. This is in sharp contrast to MS, where OCBs were shown to remain detectable over decades. Accordingly, repeat LP performed during remission may improve the CSF based laboratory differential diagnosis of NMO and MS substantially. In a previous study, we have shown that the polyspecific, intrathecal humoral immune response to measles, rubella, and varicella zoster (the so called 'MRZ reaction'), a highly sensitive (~90%) and possibly specific marker of classical MS, is mostly negative in NMO [39,40,63,67]. Corroborating these findings, we found a negative MRZ reaction in 11 additional patients in the present cohort. It is of note that OCBs were rare and tended to disappear over the course of disease also in seronegative patients; also the MRZ reaction was negative in all five seronegatives tested. This provides further support for the hypothesis that seronegative NMO is not simply a clinical variant of MS.

Infections have been discussed in the past both as a possible trigger of disease relapse in NMO and as a possible aetiological factor [1,68]. In our cohort, ON and myelitis were reported to have been preceded by acute infection at least once in 29.3% of the seropositive patients and in 17.9% of the seronegative cases. This is in good accordance with a study by Wingerchuk and colleagues who found a frequency of 30% in a North American mixed Caucasian/Asian cohort [1].

Both well defined co-existing autoimmune disorders as well as serological signs of co-existing autoimmunity were significantly more common in seropositive patients. Besides lupus erythematosus, Sjögren's syndrome, thyroid diseases, myasthenia gravis, or celiac disease, which have been all repeatedly observed in association with NMO spectrum disorders [54,69-75], a broad variety of other autoimmune conditions was present in our patients, including pyoderma gangrenosum, psoriasis, sarcoidosis, ankylosing spondylitis, collagenous colitis, atopic dermatitis, rheumatoid arthritis, Sharp syndrome, and vitiligo. The frequency of co-existing autoimmunity found in our seropositive NMO patients is higher than that observed in classical MS [76].

Four per cent of our patients had a history of cancer, including breast cancer, cervical cancer, rectal cancer, nasopharyngeal cancer, and skin cancer, all of whom were positive for AQP4-Ab. This is of interest because AQP4 was reported to be expressed in a variety of tumour tissues [77]. However, so far it is unknown whether seropositive NMO may occur as a paraneoplastic disorder, or whether the co-occurrence of the two conditions is a simple coincidence. Interestingly, our findings are in exact accordance with a recent North American study that had reported a frequency of cancer in NMO spectrum disorders in 5% [78]. In three additional patients, hysterectomy and/or ovarectomy was performed, though it remained unclear from the patients' records whether a tumour was present.

We recognize that there are some obvious limitations of our study. First, the study design was retrospective, as in all previous studies, and a multitude of neurological centres was involved. However, due to the low prevalence of NMO in Caucasians, prospective monocentre studies including sufficiently large patient numbers are impracticable. Moreover, the multicentre design of this study, which included more than 25 academic centres, reduces the risk of referral bias, which was acknowledged as a possible limitation by the authors of the two largest previous monocentre studies [1,36]. Second, similar to previous studies, analysis of MRI results was based upon patient records. However, this disadvantage is partly compensated for by the very high number of brain (N = 326) and spinal cord (N = 326) MRI results available for analysis. Third, as in previous studies, most patients were treated at least once with immunomodulatory or immunosuppressive agents. However, given the much more aggressive course of disease in NMO compared to MS, which requires early treatment, a longitudinal study on untreated patients with NMO cannot be performed. Moreover, AQP4-Ab has been previously shown to remain detectable in NMOSD even with strong immunosuppression. In a recent study, we found AQP4-Ab in 95/96 samples obtained from patients treated with a broad variety of immunosuppressive or immunomodulatory drugs such as azathioprine, rituximab, cyclophosphamide, IVIG, interferon beta, glatiramer acetate, steroids, or mitoxantrone [8]. Therefore, we do not believe that treatment effects played an important role in the present study with regard to stratification, though we can, of course, not completely exclude such effects. Fourth, assay sensitivity could be a potential limitation. However, 98.3% of our patients were tested using recombinant assays. Recombinant assays employing human AQP4-Ab have been repeatedly shown in independent studies to be significantly more sensitive compared to indirect immunofluorescence on rodent brain tissue [4,5,38,79], which was used in many of the previous studies. 19/19 (100%) samples from the seronegative group had remained negative at repeat testing (up to three times) or when tested in another assay (up to four methods); from the remaining patients no data on repeat testing and no samples were available due to the retrospective setting of this study. Fifth, patients with a benign long-term course are less likely to be admitted to hospital and might thus be under-represented in the present cohort. However, this type of potential bias is inherent in hospital-based studies and cannot be completely avoided. It is important in this context that all centres involved in the present study also have specialized neuroinflammatory outpatient departments and that patients were recruited among both inpatients and outpatients. Finally, the threshold for admission is low in Germany, where public healthcare is free. Accordingly, 7.4% of our patients had an EDSS of 0-1.5, i.e. no disability, at first admission.

In summary, our study provides a comprehensive overview of the clinical, MRI, and laboratory features of NMO in Caucasians and extends our knowledge of the clinical, diagnostic and prognostic impact of AQP4 antibody positive serostatus in this rare yet often devastating condition.

## Abbreviations

AQP4: aquaporin-4; AQP4-Ab: aquaporin-4 antibody; CSF: cerebrospinal fluid; EDSS: expanded disability status scale; IFN-beta: interferon beta; LETM: longitudinally extensive transverse myelitis; LP: lumbar puncture; MRI: magnetic resonance imaging; MS: multiple sclerosis; NMO: neuromyelitis optica; NMOSD: neuromyelitis optica spectrum disorders; NMO-IgG: neuromyelitis optica immunoglobulin G; OCB: oligoclonal bands; ON: optic neuritis.

## Competing interests

K.P.W. is an employee of Euroimmun AG, Luebeck, Germany. The other authors declare that there are no competing interests.

## Authors' contributions statement

SJ, FP, and OA conceived and designed the study; all authors were involved in patient management and acquisition of data; SJ and FP created the database; SJ analysed the data and wrote the draft; all authors took part in revising the manuscript for important intellectual content; all authors have given final approval for publication.
